# Objectively characterizing Huntington’s disease using a novel upper limb dexterity test

**DOI:** 10.1007/s00415-020-10375-8

**Published:** 2021-02-08

**Authors:** Samuel Woodgate, Philippa Morgan-Jones, Susanne Clinch, Cheney Drew, Rebecca Playle, Mohamed Bennasar, Yulia Hicks, Catherine Holt, Anne-Catherine Bachoud-Lévi, Renaud Massart, David Craufurd, Nigel Kirby, Katy Hamana, Robin Schubert, Ralf Reilmann, Anne Rosser, Monica Busse

**Affiliations:** 1 Cardiff School of Engineering, Cardiff University, Cardiff, United Kingdom; 2 NMRI, School of Medicine, Cardiff University, Cardiff, United Kingdom; 3 Centre for Trials Research, Cardiff University, 4th Floor, Neuadd Meirionnydd, Heath Park, Cardiff, CF14 4YS United Kingdom; 4 Brain Repair Group, School of Biosciences, Cardiff University, Cardiff, United Kingdom; 5 School of Computing and Communications, The Open University, Milton Keynes, United Kingdom; 6 Assistance Publique -Hopitaux de Paris, National Centre of Reference for Huntington’s Disease, Neurology Department Henri Mondor Hospital, Creteil, France; 7 Manchester Centre for Genomic Medicine, St Mary’s Hospital, Manchester University NHS Foundation Trust, Manchester Academic Health Science Centre, Manchester, UK; 8 Division of Evolution and Genomic Sciences, School of Biological Sciences, Faculty of Biology, Medicine and Health, University of Manchester, Manchester Academic Health Science Centre, Manchester, UK; 9 School of Healthcare Sciences, Cardiff University, Cardiff, United Kingdom; 10 George-Huntington-Institute, Technology-Park, Muenster, Germany; 11 Department of Clinical Radiology, University of Muenster, Muenster, Germany; 12 Department of Neurodegeneration and Hertie Institute for Clinical Brain Research, University of Tuebingen, Tuebingen, Germany; 13 INSERM U955 01, Institut Mondor de Recherche Biomédicale, UPEC, Créteil-Ecole Normale Supérieure, PSL, Paris, France

**Keywords:** Huntington’s disease, Outcome measure, Upper-limb function, Convergent validity, Regression

## Abstract

**Background:**

The Clinch Token Transfer Test (C3t) is a bi-manual coin transfer task that incorporates cognitive tasks to add complexity. This study explored the concurrent and convergent validity of the C3t as a simple, objective assessment of impairment that is reflective of disease severity in Huntington’s, that is not reliant on clinical expertise for administration.

**Methods:**

One-hundred-and-five participants presenting with pre-manifest (*n* = 16) or manifest (TFC-Stage-1 *n* = 39; TFC-Stage-2 *n* = 43; TFC-Stage-3 *n* = 7) Huntington’s disease completed the Unified Huntington’s Disease Rating Scale and the C3t at baseline. Of these, thirty-three were followed up after 12 months. Regression was used to estimate baseline individual and composite clinical scores (including cognitive, motor, and functional ability) using baseline C3t scores. Correlations between C3t and clinical scores were assessed using Spearman’s R and visually inspected in relation to disease severity using scatterplots. Effect size over 12 months provided an indication of longitudinal behaviour of the C3t in relation to clinical measures.

**Results:**

Baseline C3t scores predicted baseline clinical scores to within 9–13% accuracy, being associated with individual and composite clinical scores. Changes in C3t scores over 12 months were small ($\Omega$ ≤ 0.15) and mirrored the change in clinical scores.

**Conclusion:**

The C3t demonstrates promise as a simple, easy to administer, objective outcome measure capable of predicting impairment that is reflective of Huntington’s disease severity and offers a viable solution to support remote clinical monitoring. It may also offer utility as a screening tool for recruitment to clinical trials given preliminary indications of association with the prognostic index normed for Huntington’s disease.

**Supplementary Information:**

The online version contains supplementary material available at 10.1007/s00415-020-10375-8.

## Introduction

Huntington’s disease (HD) is an autosomal dominant neurodegenerative disorder that affects 6–13/100,000 in the general population [1]. HD is caused by the expansion of a cytosine-adenine-guanine (CAG) triplet repeat expansion on the Huntingtin gene which leads to pathological symptoms once the repeats reach thirty-six or more.

HD is characterized by a complex presentation of motor, cognitive, behavioral and functional impairments [2], and results in a progressive decline in quality of life over 15–20 years, ultimately leading to early death. Whilst there is currently no recognized cure, substantial progress is being made in disease modifying therapeutic interventions, with clinical trials underway [3]. With a real opportunity to slow functional decline on the horizon, there is an ever-increasing urgency for clinical endpoints that can truly determine the influence an intervention has on clinical progression.

At present, clinical trials typically rely on clinician-reported assessments, with the Unified Huntington’s Disease Rating Scale (UHDRS) routinely used to assess clinical performance and capacity in HD [4]. Individual components within the UHDRS facilitate the assessment of motor function, cognitive function, behavior and functional capacity [5, 6]. Whilst assessments of individual domains have furthered understanding of specific aspects of disease progression [7–9], pre-existing scales are thought to lack sensitivity to change and are prone to ceiling affects in pre-manifest and early HD [10]. There also remains a distinct lack of validated clinical tools capable of assessing upper limb function in HD [11].^.^ Such assessments are needed when seeking to understand the real life implications that a movement disorder such as HD has on activities of daily living [12]. Furthermore, the current COVID-19 pandemic has highlighted a heavy reliance on face to face assessments and a real need for robust tools that allow continued remote monitoring of symptoms when face to face clinic visits prove challenging. Subsequently, there is a recognized need for more granular and novel assessments of clinical symptoms, that can be performed in the clinic and home environments, to better evaluate the influence of novel interventions in HD.

The Moneybox test (herein referred to as the Clinch Token Transfer Test (C3t)) is a bi-manual upper limb token transfer and manipulation performance assessment, with a view to providing an ecologically valid functional assessment underpinned by sound biological rationale for people with basal ganglia dysfunction [13]. The C3t has the benefits of being quick to perform and objective in nature, with no reliance on clinical expertise to administer and thus real potential to deliver a remote solution, for monitoring clinical symptoms at scale. This in turn would increase the depth of information available to researchers to help identify and understand subtle changes in disease progression.

Early validation work found the C3t to be sensitive to HD disease stage and correlated to the components of the UHDRS and health related quality of life [13]. An instrumented version of the test, involving accelerometer data from devices worn on each wrist and the sternum, has also successfully discriminated between healthy and HD participants, with C3t derived scores highly correlated to upper-body clinician rated motor symptoms [14]. Whilst extremely promising, investigations are now required into how well the C3t can predict clinical symptoms to further evaluate its utility as an outcome measure. Additionally, work needs to be extended to investigate how well the C3t scores correlate to composite clinical scores given the multifaceted presentation of HD symptoms. Two notable composite scores requiring investigation are i) the composite UHDRS (cUHDRS) which is thought to be a stronger global indicator of disease progression, capturing the changes occurring simultaneously across the individual symptom domains in HD [15] and ii) the Prognostic Index normed for HD (PIN_HD_) [16] which has been developed to determine projected disease progression.

This study subsequently explored the concurrent and convergent validity of the C3t as a simple, objective assessment of impairment that is reflective of disease severity in Huntington’s.

## Methods

### Recruitment and governance

Data presented here were drawn from datasets across four different studies—PACE-HD, CAPIT-HD2, TRIDENT and Developing Clinical Applications for a Novel Multi-Task Functional Assessment: The Clinch Token Transfer Test (referred to here as NOVELFA-C3T).

PACE-HD (Clinical trials registration: NCT03344601) is a multi-center trial with sites in Germany, Spain, and USA, where all participants were also concurrently participating in Enroll-HD. As PACE-HD is an ongoing intervention study, only baseline data were included.

CAPIT-HD2 was a multi-center study with data collected across 4 different sites in Europe (Cardiff and Manchester, UK; Créteil Paris, France; Muenster, Germany). In the Cardiff, Manchester and Muenster sites, participants were recruited from those currently participating in the global Enroll-HD study (https://www.enroll-hd.org/). In Créteil, participants were recruited from those currently enrolled in the Predictive Biomarkers for Huntington’s disease study (Clinical trials registration: NCT01412125). Participants completed a battery of assessments during a baseline visit and were then invited to return for 1-month and 12-month visits to control for a retest effect [17]. Data collated at the 1-month timepoint involved a reduced battery of assessments (i.e., did not reflect the minimum dataset required for analyses) and was thus omitted from the analysis reported here.

TRIDENT and NOVELFA-C3T are both single-site studies based in Cardiff. Participants were recruited from those currently participating in the global Enroll-HD study. They were invited to attend a single baseline visit to complete the requisite test battery. Ethical approval for all studies was granted by Health and Care Research Wales (CAPIT-HD2 REC: 17/WA/0014, TRIDENT REC: 18/WA/0182, NOVELFA-C3T REC: 17/WA/0014).

All participants included in the studies were 18 or more years of age, with genetically confirmed HD and the capacity to provide informed consent. Diagnosis of HD was categorized into one of four disease stages at each visit (Pre-manifest, Diagnostic Confidence Interval (DCI) ≤ 3; Stage 1, Total Functional Capacity (TFC) = 11–13 and DCI = 4; Stage 2, TFC = 7–10; Stage 3, TFC = 4–6) [15]. The CAG Age Product score (CAP) [18] was calculated using age and CAG repeat length to estimate disease impact for demographic purposes [19] (Eq. 1).

1 $$\mathrm{CAP}=\mathrm{Age}*\frac{\mathrm{CAG}-30}{6.49}$$

### Assessments and outcome measures

All participants (*n* = 105) performed the C3t or a prior version of the task (Moneybox Test (MBT)) at the baseline visit with a subset repeating this at 12 months (*n* = 33). Both versions of the test involve the performance of six (C3t) or five (MTB) tasks, with this study focusing on two of the tasks performed identically across test versions—the Baseline Transfer Task (BTT) and Complex Transfer Task (CTT) which are described in full in the C3t manual (Supplementary Material 1). In both BTT and CTT tasks, participants picked up a token one at a time with their non-dominant hand, transferred it to their dominant hand and placed it into a slotted box. During the BTT participants transferred eight blank tokens in order of physical size (largest to smallest). During the CTT a mild cognitive load is added by asking participants to transfer a different set of tokens in order of the number printed on them (highest to lowest). In both tasks the primary measure recorded is the time taken to complete the task (i.e., transfer all tokens successfully).

Participants also completed the Unified Huntington’s Disease Rating Scale (UHDRS), with scores produced to assess the symptom domains of motor function (Total Motor Score (TMS)) and cognition (Symbol Digit Modalities Test (SDMT) and Stroop Word Reading Test (SWRT)) along with capacity (Total Functional Capacity (TFC)). The composite UHDRS (cUHDRS) [15] was used to provide a global indicator of HD disease progression (Eq. 2).

2 $${\text{cUHDRS}} = \left[ {\left( {\frac{{{\text{TFC}} - 10.4}}{{1.9}}} \right) - \left( {\frac{{{\text{TMS}} - 29.7}}{{14.9}}} \right) + \left( {\frac{{{\text{SDMT}} - 28.4}}{{11.3}}} \right) + ~\left( {\frac{{{\text{SWR}} - 66.1}}{{20.1}}} \right)} \right] + 10$$

Scores on the select UHDRS assessments (TMS, SDMT, SWRT, TFC) and the cUHDRS were used as criterion clinical measures to assess the convergent and concurrent validity of the C3t.

The prognostic index normed for HD (PIN_HD_) [16] was calculated (Eq. 3) to determine projected disease progression in the pre-manifest subgroup, with higher scores indicating greater risk of motor diagnosis. A PIN_HD_ of less than 0 indicates greater than 50% 10-year survival, whilst a PIN_HD_ of greater than 0 indicated less than 50% 10-year survival.

$${\mathrm{PIN}}_{\mathrm{HD}}= \frac{{\mathrm{PI}}_{\mathrm{HD}}-883}{1044}$$

where

3 $${\mathrm{PI}}_{\mathrm{HD}}= 51 \times \mathrm{TMS}+\left(-34\right)\times \mathrm{SDMT}+7 \times \mathrm{Age} \times (\mathrm{CAG}-34)$$

All data were stored in a SQL database using Python (v3.7) and subsequently analyzed using SciPy (v1.3.0) [20].

### Data analysis

The C3t time taken BTT and CTT scores were assessed for normality using multiple statistical tests (Shapiro-Wilks Test, D’Agostino K-Squared Test and Anderson–Darling Test) and visually inspected using histograms and Q-Q plots. Given their non-normal distribution, non-parametric data analysis methods were employed.

Least squares regression was performed to determine whether HD disease severity, as measured by the UHDRS (cUHDRS, TMS, SMDT and SWRT), could be predicted using C3t scores (BTT and CTT). The coefficients from a LASSO regression (see Supplementary Material 2) confirmed that data collected across sites and using the two different versions of the C3t could be pooled as both were found to have negligible effects on the regression model. Scatterplots of C3t time scores (BTT and CTT) plotted against the TMS, SDMT, SWRT and cUHDRS revealed a non-linear relationship and as such a polynomial regression (degree of 2) was performed to optimize the predictive model. The TFC was not predicted due to a lack of any discernable pattern with the C3t scores (which was confirmed via a scatterplot of C3t time scores (BTT and CTT) plotted against TFC). The PIN_HD_ was also not predicted due to insufficient sample size as this could only be measured for the pre-manifest sub-group (*n* = 16). Only data collected during the baseline visit were used in the regression models. To ensure robust results, repeated *k*-fold cross-validation was used. Cross-validation is a common method used in machine learning to avoid overfitting statistical models to datasets, helping to ensure robust, generalizable results. *K*-fold cross-validation splits the dataset into *k* partitions, withholding one partition and training/constructing a regression model using the remaining *k*-1 partitions. The quality of the trained regression model is then assessed using the withheld partition. This process is conducted k times, with each partition taking a turn at being withheld and used to assess a regression model trained using the remaining *k*-1 partitions. In repeated k-fold cross-validation this process is then repeated by randomly shuffling the original dataset. Doing so is designed to ensure the internal structure of the original dataset has not, by chance, influenced the results of the models.

In this study four folds and ten repeats were used (*k* = 4, repeats = 10), resulting in 40 models constructed overall. During each cross-validation fold, the ratio of TFC Stages present in the entire dataset was maintained in the training and testing sets. The mean absolute error (MAE) and the normalized MAE were used to assess model quality for each of the 40 models generated during the cross-validation process (Eq. 4). The mean MAE and normalized MAE across the 40 models are reported. The MAE quantifies on average how far off from the actual value (regardless of direction) a model is, across a population when predicting a dependent variable (in this case the UHDRS scores). The normalized MAE was utilized to allow comparison across outcomes with varying ranges:

4 $$\mathrm{MAE}=\frac{{\sum }_{i=1}^{n}|{y}_{i}-{x}_{i}|}{n}$$

where *y*_*i*_ is the actual clinical score, *x*_*i*_ is the predicted score and n is the sample size.

The strength and direction of association between each clinical score and the C3t scores was measured using Spearman’s *R* correlation coefficient. A-priori statistical level of significance was set to *p* ≤0.05.

Scatterplots are reported for each clinical score in relation to the BTT and CTT scores, with a key denoting disease stage for each participant. These were visually inspected to evaluate the sensitivity of the C3t to measure clinical impairment across the spectrum of disease state. PIN_HD_ was plotted in a similar manner against the BTT and CTT scores to investigate how sensitive the C3t scores may be to overall risk of motor diagnosis and probability of 10-year survival. With the PIN_HD_ designed to estimate progression levels in pre-diagnosis HD, this was only performed on the pre-manifest sub-group.

Baseline to 12-month changes in C3t scores were assessed using effect size and compared to clinical scores using data from all participants, where follow-up data were available (*n* = 33, with 11 lacking a SWRT score thus reducing sample size to 22 when measuring change in SWRT and cUHDRS). Effect size was calculated using a nonparametric analog of Cohen’s *D*, omega (Ω), where Ω = 0 indicates no effect and values of ± 0.1, ± 0.3 and ± 0.4 correspond to the descriptors used for Cohen’s *D* indicating low, medium and large effect sizes, respectively [21]. Unlike Cohen’s *D*, Ω is directional and as such for the sake of simplicity the absolute value of *Ω* is reported throughout.

## Results

### Participants

One-hundred-and-five gene-positive participants were recruited at the baseline visit across all studies and sites (see Table 1**,** with the number of participants recruited across each sub-study and disease stage reported in Supplementary Material 3). Thirty-three participants also attended the 12-month follow-up visit.

**Table 1:** Participant demographics (mean ± (standard deviation)) at the baseline visit subdivided by TFC Stage group and analysis stage

Analysis stage	TFC stage group	*n*	Age	% Female	CAG	CAP	UHDRS-TFC	UHDRS-TMS	SDMT	SWRT	PIN	CUHDRS
Correlation / Regression Analysis (Whole Cohort)	Pre-manifest	16	46.1 (± 11.3)	31.2	42.4 (± 1.8)	88.2 (± 25.7)	12.2 (± 1.5)	7.6 (± 6.8)	46.4 (± 13.2)	87.6 (± 18.4)	0.6 (± 1.4)	15.1 (± 2.8)
	TFC Stage 1	39	54.4 (± 11.2)	25.6	42.9 (± 2.4)	105.0 (± 14.5)	12.0 (± 0.8)	25.2 (± 12.0)	34.9 (± 9.5)	71.5 (± 15.0)	2.4 (± 1.2)	12.0 (± 2.0)
	TFC Stage 2	43	52.7 (± 11.8)	51.2	44.2 (± 4.3)	109.0 (± 14.7)	8.8 (± 1.1)	39.4 (± 12.5)	23.7 (± 9.4)	56.7 (± 14.7)	3.6 (± 1.1)	7.6 (± 2.1)
	TFC Stage 3	7	46.1 (± 8.3)	28.6	43.9 (± 1.0)	98.5 (± 18.3)	5.3 (± 0.5)	43.7 (± 20.5)	20.3 (± 9.6)	46.0 (± 9.6)	3.7 (± 1.7)	4.6 (± 2.3)
	Total	105	51.9 (± 11.7)	37.1	43.4 (± 3.3)	103.7 (± 18.4)	10.3 (± 2.3)	29.6 (± 17.0)	31.1 (± 13.2)	66.2 (± 19.3)	2.7 (± 1.6)	10.2 (± 3.8)
Effect Size: Baseline—12 months (excluding SWRT & cUHDRS)	Pre-manifest	3	55.3 (± 5.8)	33.3	42.3 (± 2.1)	104.3 (± 15.0)	12.3 (± 0.5)	3.7 (± 3.8)	38.7 (± 9.9)	83.3 (± 15.3)	1.1 (± 0.9)	14.5 (± 1.6)
	TFC Stage 1	15	53.5 (± 12.4)	40	43.2 (± 2.2)	104.8 (± 13.8)	11.6 (± 0.7)	27.7 (± 12.9)	32.7 (± 9.5)	68.0 (± 13.0)	2.6 (± 1.1)	11.2 (± 2.1)
	TFC Stage 2	14	49.7 (± 11.1)	35.7	45.1 (± 4.2)	109.4 (± 12.6)	9.0 (± 1.1)	41.2 (± 13.2)	23.9 (± 10.2)	57.8 (± 14.8)	3.8 (± 1.1)	7.7 (± 2.5)
	TFC Stage 3	1	53.0 (± 0.0)	100	43.0 (± 0.0)	106.2 (± 0.0)	6.0 (± 0.0)	32.0 (± 0.0)	19.0 (± 0.0)	N/A	3.3 (± 0.0)	N/A
	Total	33	52.0 (± 11.4)	39.4	43.9 (± 3.3)	106.8 (± 13.4)	10.4 (± 1.8)	31.4 (± 16.3)	29.1 (± 11.1)	65.5 (± 16.2)	3.0 (± 1.3)	10.1 (± 3.2)
Effect Size: Baseline—12 months (including SWRT and cUHDRS)	Pre-manifest	3	55.3 (± 5.8)	33.3	42.3 (± 2.1)	104.3 (± 15.0)	12.3 (± 0.5)	3.7 (± 3.8)	38.7 (± 9.9)	83.3 (± 15.3)	1.1 (± 0.9)	14.5 (± 1.6)
	TFC Stage 1	8	59.1 (± 7.6)	50	42.5 (± 1.5)	112.3 (± 6.4)	11.4 (± 0.7)	28.5 (± 9.1)	29.0 (± 9.0)	65.8 (± 12.4)	2.9 (± 0.8)	10.6 (± 1.7)
	TFC Stage 2	11	47.9 (± 9.2)	45.5	45.2 (± 3.9)	107.6 (± 12.3)	8.9 (± 1.2)	40.5 (± 14.0)	24.0 (± 10.7)	57.8 (± 14.8)	3.7 (± 1.2)	7.7 (± 2.5)
	TFC Stage 3	0	N/A	N/A	N/A	N/A	N/A	N/A	N/A	N/A	N/A	N/A
	Total	22	53.0 (± 9.8)	45.5	43.8 (± 3.3)	108.9 (± 11.4)	10.3 (± 1.7)	31.1 (± 16.7)	27.8 (± 11.1)	64.2 (± 16.4)	3.1 (± 1.3)	9.7 (± 3.2)

### C3t scores in relation to UHDRS clinical scores

**Fig. 1:**
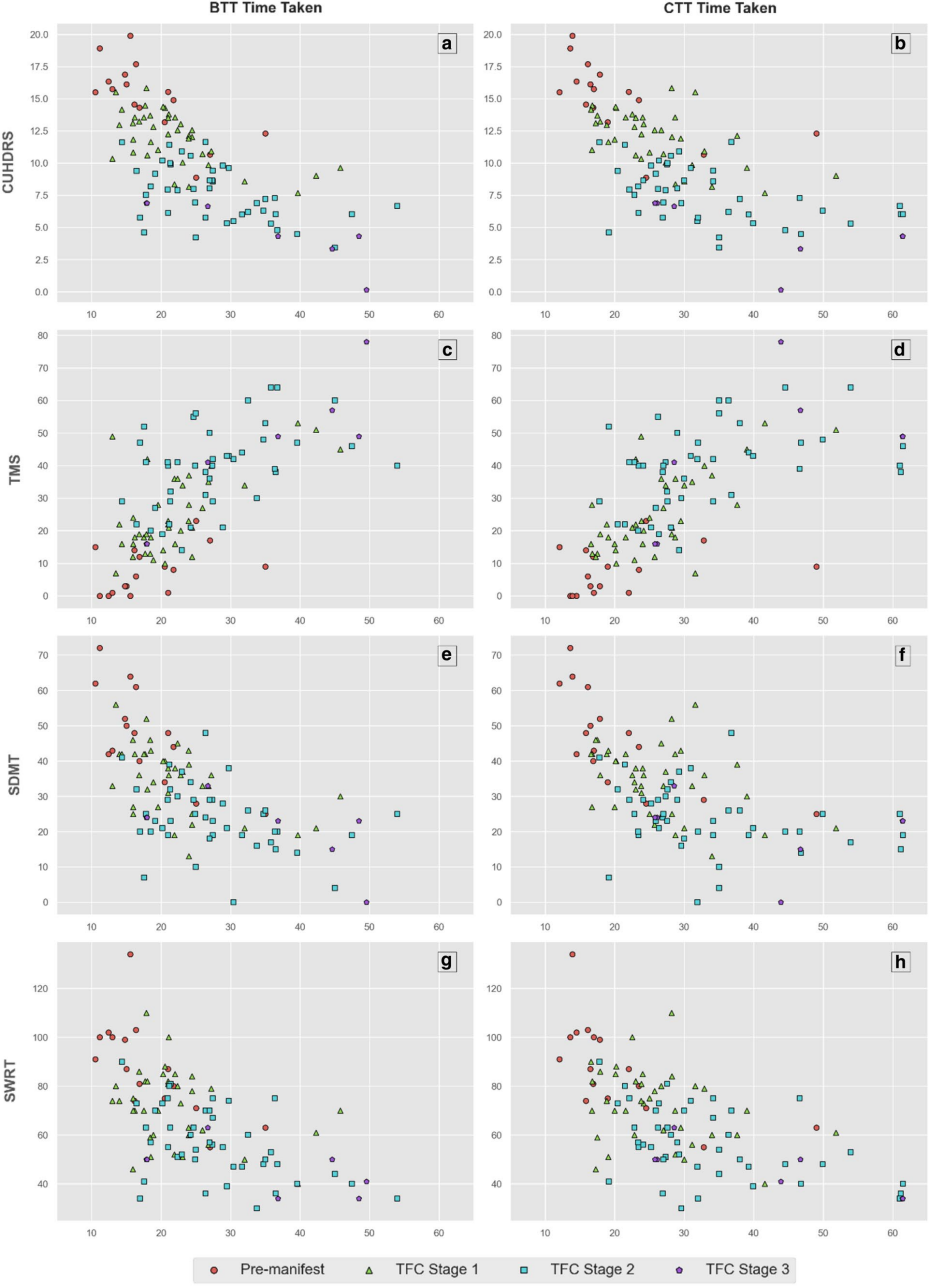
Time taken to perform the Baseline Transfer Task (BTT) and Complex Transfer Task (CTT) plotted against the Composite Unified Huntington’s Disease Rating Scale (cUHDRS), Total Motor Score (UHDRS-TMS), Symbol Digit Modalities Test Number Correct (SDMT) and Stroop Word Reading Test (SWRT). Participant disease stage is denoted by the key

**Fig. 2:**
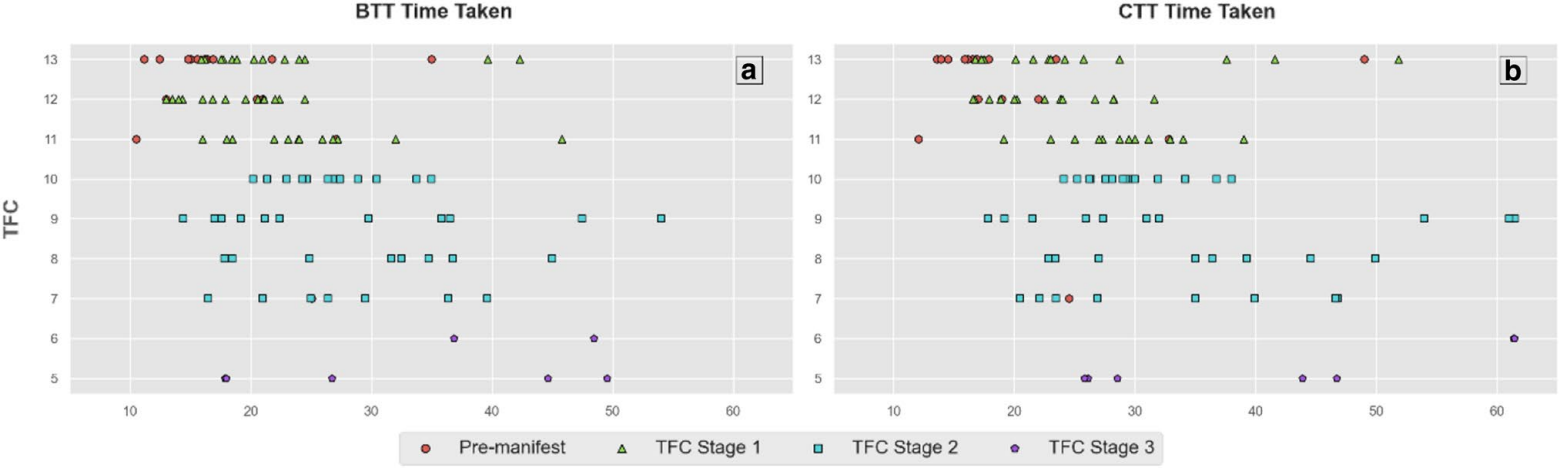
Time taken to perform the Baseline Transfer Task (BTT) and Complex Transfer Task (CTT) plotted against the Total Functional Capacity (TFC) with disease stage of each participant denoted by the key

C3t time scores and each of the four UHDRS measures during the baseline were highly and significantly associated (*p* < 0.001) with one another (see Table 2) with the strongest correlation identified between the cUHDRS and the CTT time scores (CTT *r* = − 0.7). A positive association was found between time taken to perform both C3t tasks and TMS, where BTT and CTT time increased as TMS score increased. In contrast, negative associations were found for the cUHDRS, SMDT and SWRT, where time taken to perform BTT and CTT increased as clinical scores decreased. Scatterplots plotting each clinical score under analysis against the BTT and CTT C3t scores are presented in Fig. 1 to visually represent these associations (with a key denoting disease stage for each participant to aid contextualization). No further investigation of relationships between C3t and TFC were indicated given the lack of any association between C3t scores and the TFC (see Fig. 2).When estimating baseline clinical scores using baseline C3t scores, normalized mean absolute error ranged from at best 9% and at worst 13% (see Table 2).

**Table 2:** Regression results for C3t scores and clinical measures at baseline

C3t score	Clinical score	Spearman’s *R*	Mean absolute error (± SD)	Normalised mean absolute error	Cohort max clinical value
Baseline transfer task time taken	cUHDRS	− 0.69**	2.23 (± 0.33)	11.0% (± 2.0%)	19.89
	TMS	0.67**	9.88 (± 1.43)	13.0% (± 2.0%)	78
	SMDT	− 0.63**	8.14 (± 1.08)	11.0% (± 2.0%)	72
	SWRT	− 0.62**	12.05 (± 1.8)	9.0% (± 1.0%)	134
Complex transfer task time taken	cUHDRS	− 0.7**	2.11 (± 0.3)	11.0% (± 1.0%)	19.89
	TMS	0.69**	9.4 (± 1.18)	12.0% (± 2.0%)	78
	SMDT	− 0.64**	7.63 (± 1.15)	11.0% (± 2.0%)	72
	SWRT	− 0.64**	12.02 (± 1.47)	9.0% (± 1.0%)	134

Spearman’s *R* (** indicates *p* < 0.001), its corresponding *p*-value, the Mean Absolute Error (MAE) and Normalised MAE are reported. Spearman’s *R* is calculated across the entire baseline dataset. The MAE and Normalised MAE are the mean results from repeated, *k*-fold cross-validated models (*k* = 4, repeats = 100) stratified across TFC stages. MAE and Normalised MAE results are the mean (± standard deviation) across all folds. Cohort max is defined as the maximum value for each clinical measure in the dataset

### C3t scores in relation to predicted 10-year survival rate and motor diagnosis

**Fig. 3:**
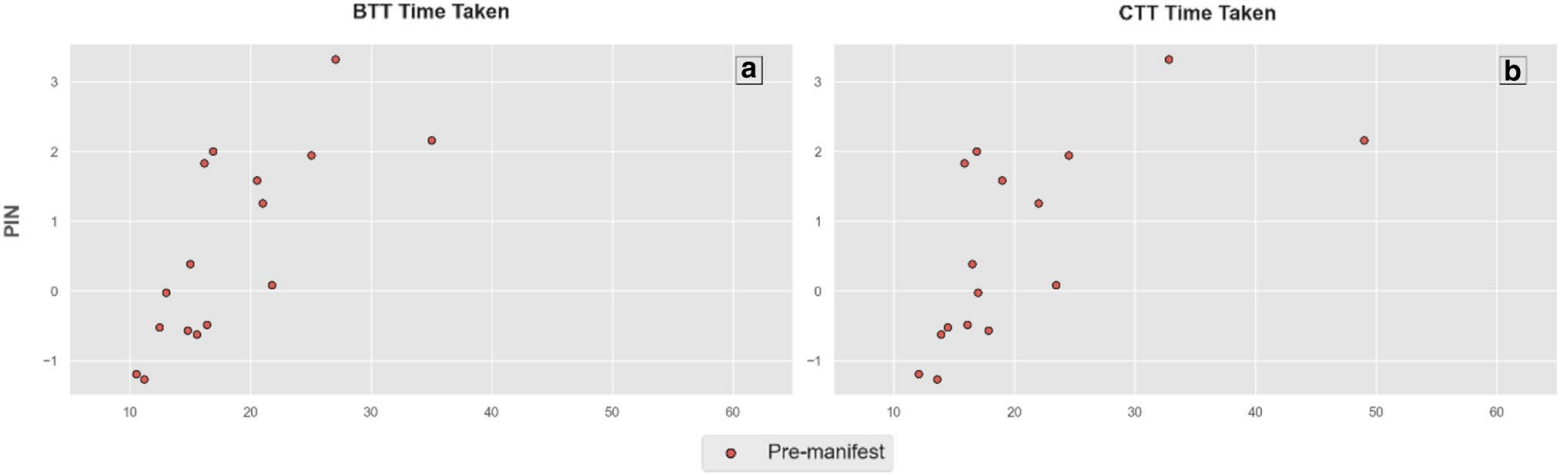
Time taken to perform the Baseline Transfer Task (BTT) and Complex Transfer Task (CTT) plotted against Prognostic Index for HD (PIN_HD_) for pre-manifest participants

A positive correlation was found between timed taken to perform both C3t tasks and PIN_HD_ in the pre-manifest sub-group (BTT *r* = 0.83, *p* < 0.001; CTT *r* = 0.76, *P* < 0.05), where BTT and CTT increased as PIN_HD_ score increased (see Fig. 3).

### Assessing the 12-month behavior of the C3t

Small changes in C3t scores were reported for baseline to 12-month changes ($\Omega$ ≤ 0.15) which mirrored the small change reported in all clinical scores (see Table 3).

**Table 3:** Effect size, *Ω*, (non-parametric analogue of Cohen's *D*) results for baseline to 12 months

	Baseline to 12-month effect size ($\Omega$)
Baseline Transfer Task (BTT)	− 0.060
Complex Transfer Task (CTT)	0.100
Total Motor Score (TMS)	− 0.073
Symbol Digit Modalities Test (SDMT)	0.115
Stroop Word Reading Test (SWRT)	0.112
Unified Huntington’s Disease Rating Scale (cUHDRS)	0.062

Due to incomplete data, the sample size for SWRT and cUHDRS effect size was reduced from *n* = 33 to *n* = 22

## Discussion

The C3t is a quick, easy to administer objective assessment that is associated with HD disease status. Previous work has shown that the time to perform C3t tasks is related to increasing disease manifestation [13]. Here we have re-confirmed these findings on a significantly larger cohort with representation across a broader spectrum of disease. Additionally, we have shown that the BTT and CTT task times can be used to predict gold-standard motor, cognitive and composite clinical measures with a high-degree of accuracy.

In the original development of the C3t, it was proposed that the dual task paradigm would elicit motor impairment perhaps not seen in simple or single task conditions [22]. Yet, it appears that time taken to perform the simple task (BTT) and dual task (CTT) are correlated with both motor and cognitive clinical outcomes (i.e., increased time to complete the C3t tasks may be as a result of cognitive or motor deficits or a combination of both). Whilst it is possible that individuals with HD could exhibit differing prioritization strategies based on cognitive and motor ability, previous studies have found that the majority of participants exhibited mutual interference or prioritized motor over cognitive activities [23]. With the BTT and CTT analyses demonstrating comparable findings, we suggest that there is limited added value in performing both the BTT and CTT when seeking to estimate global clinical progression using the time taken to perform a given task. The utility of each task may, however, lie beyond these simple temporal measures and requires further investigation.

The notion of multiple domains influencing clinical outcomes has been identified by Schobel [15] who recommended the use of a global measure of clinical disease progression in the cUHDRS given the multifaceted presentation of HD. As a composite product of the UHDRS, the cUHDRS combines cognitive, motor, and global functional symptom domains, and has been found to have enhanced sensitivity to clinical change in early symptomatic HD [9]. The C3t timing measures were highly associated and predicted the cUHDRS to within 11% of the actual score.

C3t scores were found to be associated to PIN_HD_ in pre-manifest individuals, with a trend for increased time to perform the C3t in people with higher PIN_HD_ scores. Thus, it appears that the C3t scores are capable of measuring symptoms in relation to predicted 10-year survival rate and motor diagnosis. This in part would appear to be linked to the role that both the TMS and SDMT play in the calculation of PIN_HD_ as these scores have independently been found to be associated with the C3t. Given the proposed utility of the PIN_HD_ to assist the identification of suitable participants into HD clinical trials [16], the C3t shows promise as a recruitment screening tool that could lead to improved efficiency in clinical trials. With pre-manifest participants representing only a relatively small subgroup of the current study cohort, further investigation is, however, needed involving a larger pre-manifest cohort.

No association between the C3t scores and function as measured by TFC was found. TFC is routinely used to define the stages of disease severity. It provides a standardized scale to assess capacity to work, deal with personal finances, perform domestic chores and activities of daily living and self-care tasks. As such, many factors contribute to functional decline that is reflected by TFC scores. In contrast, the C3t is designed to primarily assess motor symptoms and has the potential to be impacted by cognitive impairment (e.g., psychomotor slowing and attentional deficits). As such, whilst some relationship might be expected, the two assessments are focused on different measurement domains.

Small changes in C3t scores over a 12-month period were in line with those seen in clinical outcomes over the same duration. Whilst greater changes in UHDRS over 12 months have been reported previously [15, 24], progression of clinical symptoms can be highly varied within the cohort under investigation. The ability to mirror changes in clinical outcomes is positive but further investigation is needed to ensure this is replicated when larger clinical progression is present. Investigations into the short-term stability of the C3t when clinical symptoms remain stable is also warranted and will be the focus of future work.

A recognized limitation of this study is the under representation of later stage and pre-manifest participants. Surprisingly, later stage participants (Stage 3) were younger than those in earlier disease stages. We believe this is most likely a chance finding given the small numbers of late stage participants in our cohort (*n* = 7). Future work should focus on larger sample sizes and in particular the representation of both pre-manifest and later stage participants. Larger sample sizes per sub-group will allow further analysis to establish whether the predictive performance of the C3t is stronger in a particular disease stage.

In conclusion, the time taken to perform the C3t tasks is associated and reasonably predictive of HD disease status as assessed by the UHDRS. Being associated with PIN_HD_ in pre-manifest participants, it may also offer utility as a screening tool for recruiting onto clinical trials. Furthermore, the C3t scores mirrored the small changes in clinical impairment over 12 months. This study supports the potential utility of the C3t to objectively estimate global clinical symptom severity in HD. It requires minimal equipment, time, and clinical expertise to perform, thus offering a viable solution for remote monitoring of clinical impairment. To facilitate such monitoring, emphasis now needs to be placed on streamlining the way in which the C3t can be performed in the home setting and how the data is returned to the clinical team. With the ability of sensor integration, the C3t also facilitates the collection of detailed upper limb movement during task performance [14]. Thus, enhancing its potential as a sensitive assessment of motor function in clinical trials. Given the limited sample size available for the longitudinal analysis, future studies should have a specific focus on assessing the psychometric properties of the C3t over time and across the spectrum of disease manifestation.

## Supplementary Information

Below is the link to the electronic supplementary material.

Supplementary file1 Supplementary material 1: The Clinch Token Transfer Test Manual (PDF 3760 KB)

Supplementary file2 Supplementary material 2: The coefficients reported from a LASSO regression when predicting clinical scores using either the BTT or CTT C3t scores, taking into consideration the site at which the data was collected at and the version of the C3t that was performed. The lower the magnitude of a coefficient the less impact the relevant variable has on the model. Coefficients are reported as mean and standard deviation (SD) to reflect the variation across the cross-validation folds within the regression analyses. Only the site and C3t version with the highest coefficient are reported to determine how much site and test version contributed to the regression model. Whilst some site and test version coefficients were non-zero, their magnitude was significantly smaller and more variable than the C3t time score coefficients, suggesting they had limited impact on the model relative to the C3t (DOCX 24 KB)

Supplementary file3 Supplementary material 3: The number of participants recruited across each sub-study and disease stage (DOCX 21 KB)
