# Supplementary material for: Objectively characterizing Huntington’s disease using a novel upper limb dexterity test
Source: J Neurol. 2021 Feb 8;268(7):2550–9. doi: 10.1007/s00415-020-10375-8 (PMC7868671; doi:10.1007/s00415-020-10375-8)
Supplement: Supplementary file 1 — Supplementary file1 Supplementary material 1: The Clinch Token Transfer Test Manual (PDF 3760 KB) [file 415_2020_10375_MOESM1_ESM.pdf]

THE CLINCH TOKEN TRANSFER TEST

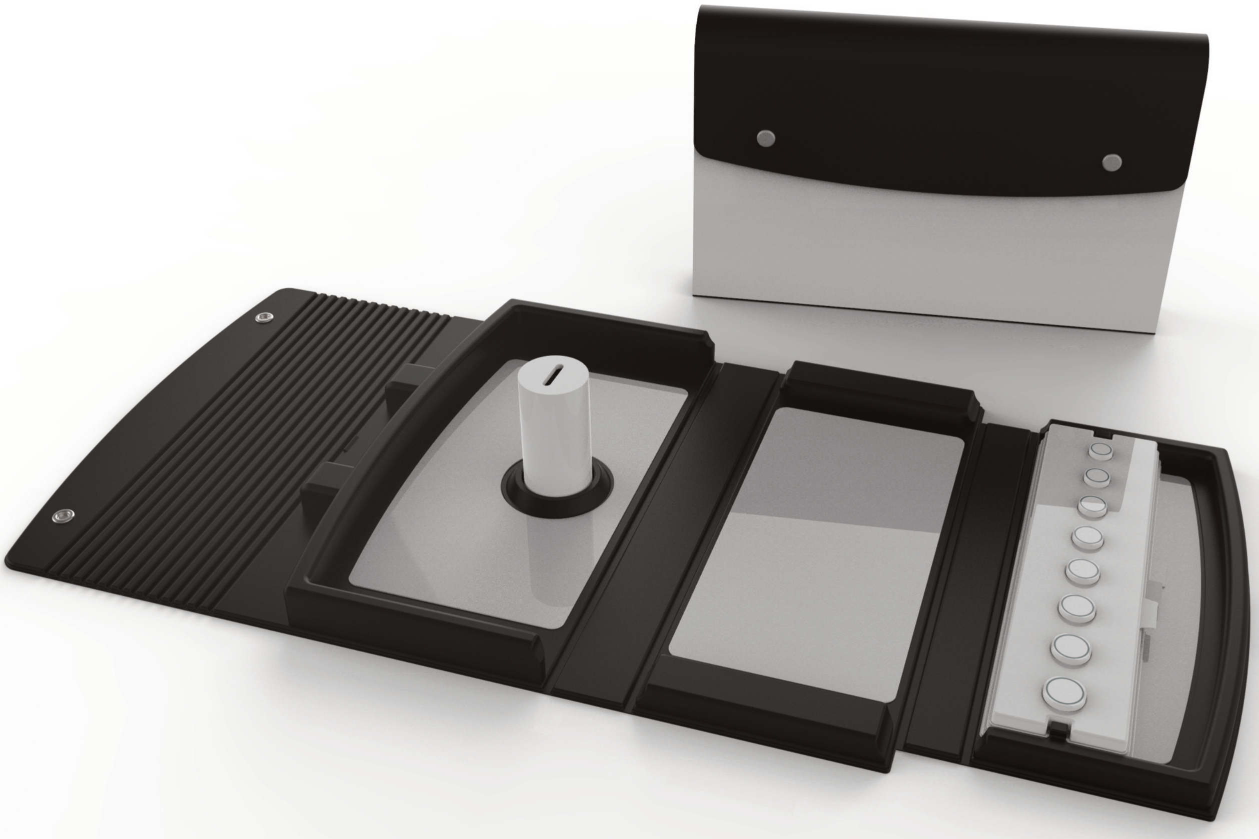

Developers: Susanne Clinch, Monica Busse, Mariah Lelos, Anne Rosser

© Cardiff University

TABLE OF CONTENTS

INTRODUCTION 2

C3T EQUIPMENT 2

TEST PROCEDURE 6

VERBAL INSTRUCTIONS FOR EACH TASK 8

1 Baseline Transfer task: 8

2 Baseline Value task 8

3 Complex Value task 8

4 Baseline Alphabet task 8

5 Complex Transfer task 8

6 Dual Transfer task 8

SCORING 9

FREQUENTLY ASKED QUESTIONS (FAQS) 11

DATA COLLECTION SHEET 12

INTRODUCTION

The Clinch token transfer test (C3t) is a brief, standardized clinical assessment tool that is used to quantify upper extremity function.

Briefly; the subject is seated at a table in front of the C3t test kit. The kit contains three sets of tokens and a purpose manufactured container.

When instructed, the subject is required to complete a series of token transfer activities. The subject is asked to pick up a token and transfer it from their non-dominant to their dominant hand. Tokens are either transferred in order of size (baseline transfer) or value, without or whilst reciting the alphabet (complex transfer and dual transfer respectively). The addition of cognitive load increases the task complexity. The time taken to pick up and transfer the tokens to the C3t container as well as the number of errors are recorded.

This manual describes the setup and testing procedures required to administer the Clinch token transfer test (C3t).

C3t EQUIPMENT

All the equipment needed to conduct the C3t is provided as part of the C3t test kit (see Figure 1). This includes:

a C3t container

b Three magnetic token trays, one for each task (Baseline, Complex and Dual tasks)

c Eight magenetic tokens, one for each task (see Figure 2):

- i. Baseline Transfer (circle indentations on rear). No value is printed on participant facing side.
- ii. Complex Transfer (diamond indentations on rear). The values 200,100,50,20,10,5,2,1 are printed on the participant facing side.
- iii. Dual Transfer (triangle indentations on rear). The values 90, 82, 71, 49, 35, 17, 6, 3 are printed on the participant facing side.

d and e) Two Baseline Value cards

e C3t case

A stop watch, table and hard back chair are required for conducting the test, but are not provided with the C3t test kit.

Figure 1: C3t test components

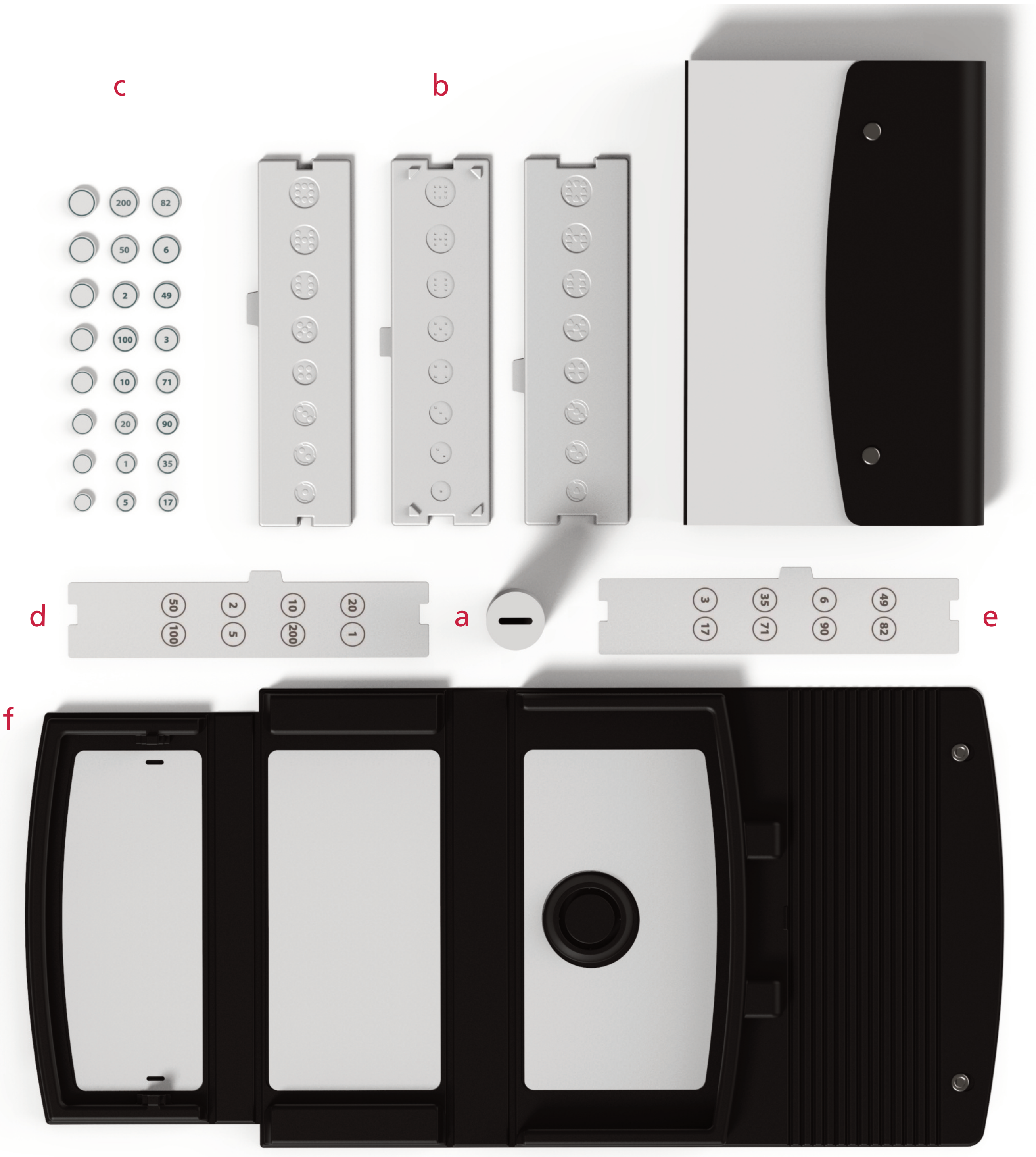

Figure 2: Token order for the Baseline Transfer, Complex Transfer and Dual Transfer tasks.

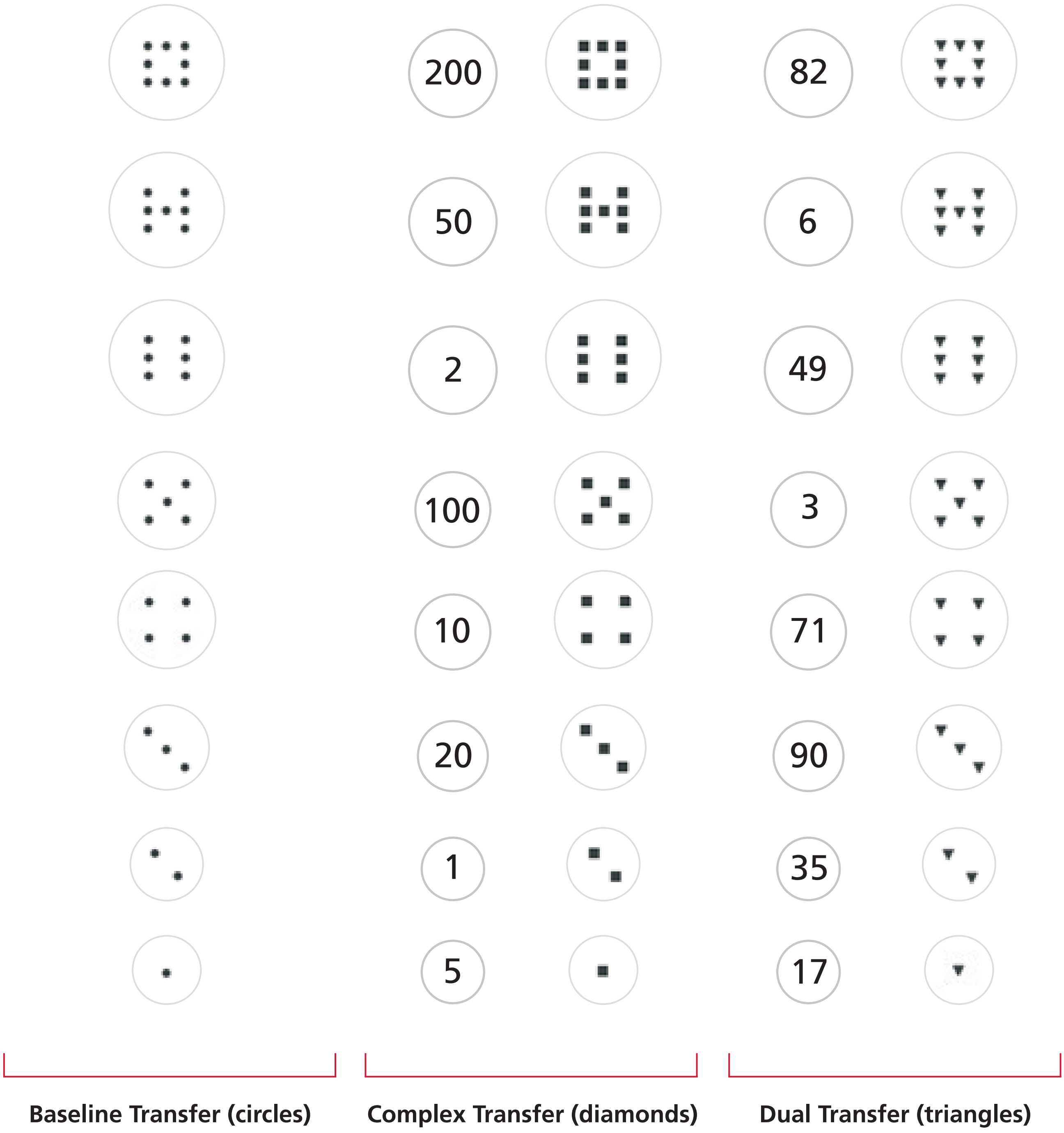

Additional guidance is available from the developers for the integration of accelerometer devices during test performance. These devices can facilitate more detailed and sensitive assessment of movement characteristics during test performance.

PROCEDURE FOR SETTING UP THE C3t EQUIPMENT

1. Lay the C3t test kit open onto a flat surface as shown in Figure 3 below.
2. If the subject is right-handed, the circular indentation should be positioned on the right side of the case. If the subject is left-handed, make sure that the circular indentation is on the left side of the case.
3. Place the C3t container upright in the circular indentation, ensuring that the slot is vertical to the participant.
4. Remove the elastic band that stretches around the trays when the C3t test kit is stored.
5. Match the circular, diamond and triangle patterned tokens with the patterned indentations on the token trays (Figure 2)
6. Stack the trays onto the stepped grooves in the C3t case, ensuring that the largest token is furthest away from the subject.

The trays should be stacked as described below:

i. Insert the tray with the triangle indents in first. This is for the Dual Transfer task.
ii. Position the tray with the diamond indents on top of the first tray. This is for the Complex Transfer task.
iii. Lay the complex value card on top (featuring numbers 49, 82, 6, 90, 35, 71, 3, 17)
iv. Lay the Baseline Value card on top of this (featuring 20, 1, 10, 200, 2, 5, 50, 100).
v. Position the tray with the tokens that have no values (circle indents) on top. This is for the Baseline Transfer task.

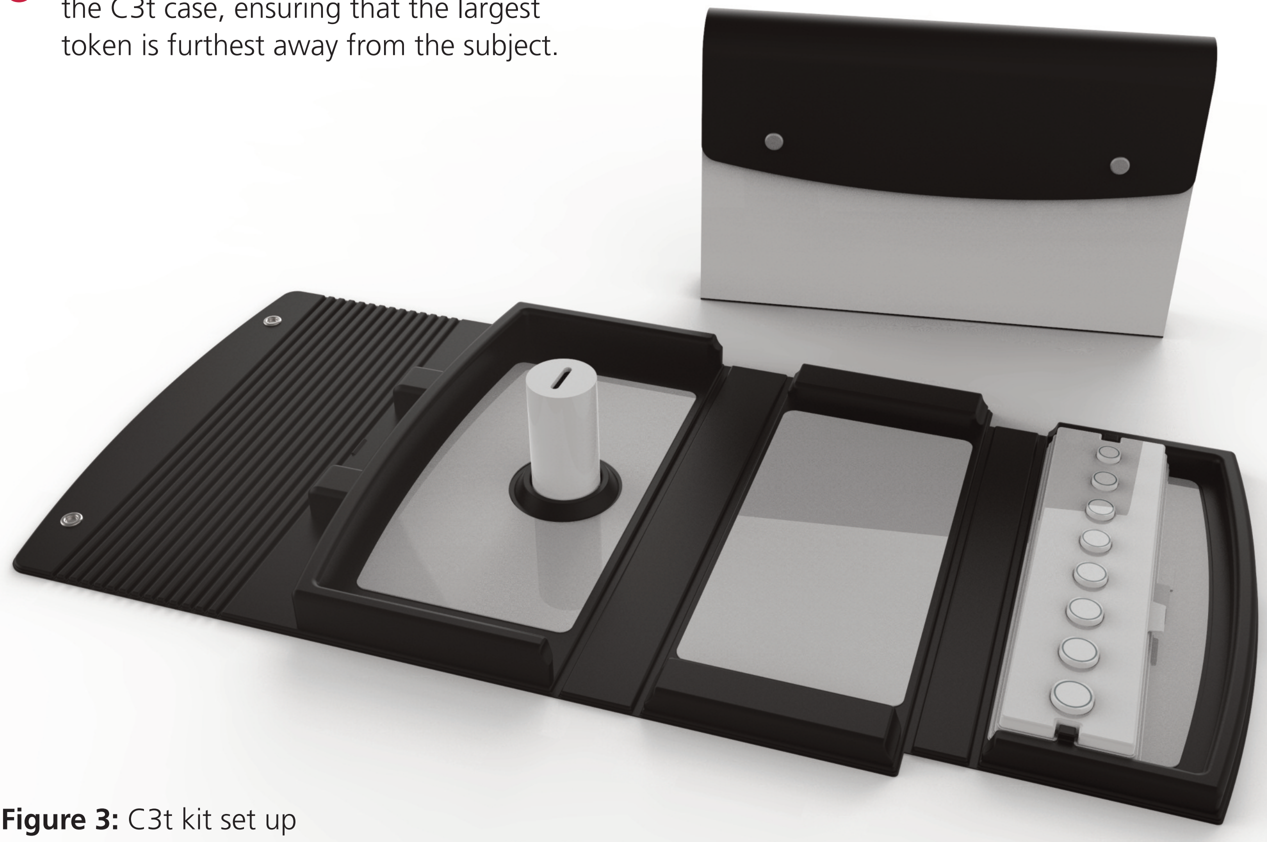

Figure 3: C3t kit set up

TEST PROCEDURE

The subject is asked to sit on a hard-backed chair with the C3t positioned in front of them on a table. Ensure that the C3t is set up correctly for either a right or left-handed subject.

The complete C3t assessment requires the subject to complete six tasks; three involving the transfer of tokens, Baseline Transfer, Complex Transfer and Dual Transfer tasks and three involving verbal tasks. A C3t scoring sheet is provided with this manual in which you should record time taken and number of errors for each of the tasks.

THE PROCEDURE AND THE C3t TASK ORDER IS DESCRIBED BELOW.

1 **Baseline Transfer task:** The subject is asked to transfer the first row of tokens, in order of size, starting with the largest token, as quickly as possible. This should be fairly simple for the subject as the tokens are already positioned in front of them in size order. The largest token should be located furthest away from the subject when they are seated at the table.

2 **Baseline Value:** The top tray is removed to reveal the baseline value card. The subject is shown eight values (20, 1, 10, 200, 2, 5, 50, 100) and asked to recite the values in numerical order from the highest to the lowest value. This test is to ensure the subject can count backwards using the same values that will be presented on the tokens in the complex transfer task. The card must remain covered whilst the test instructions are read to ensure the subject does not attempt to determine the token order before the task begins. If they are unable to complete this task, with at least 6/8 correct, the subject should not progress to the complex transfer task.

3 **Complex Value (optional, if progressing to dual task):** The baseline value card is removed to reveal the complex value card. Eight different, more complex values are presented (49, 82, 6, 90, 35, 71, 3, 17). The subject is asked to recite the values in numerical order from the highest to the lowest value. This test is to ensure the subject can count backwards using the same values that will be presented in the dual transfer task. The card must remain covered whilst the test instructions are read to ensure the subject does not attempt to determine the token order before the task begins. If you are not intending on conducting the dual transfer task test (6) then you can omit the complex value task. If they are unable to complete this task, with at least 6/8 correct, the subject should not progress to the dual transfer task (number 6).

4 **Baseline Alphabet (optional, if progressing to dual task):** The subject is asked to recite the alphabet once, as quickly as possible. The subject can recite the

alphabet in any language, as long as the researcher can record the number of correct and incorrect letters recited. This baseline test is carried out in preparation for the dual transfer task. This must be completed within 60 seconds in order to proceed with the dual task transfer. If a subject is unable to complete this task (75% correct; equating to 20/26 in the English language) or taking longer than 60 seconds, they should not progress to the dual transfer task (number 6). If you are not intending on conducting the dual transfer task test (6) then you can omit the baseline alphabet recital.

5 **Complex Transfer task:** The subject is asked to transfer the tokens in order of value from highest to lowest. *The tokens must remain covered whilst the test instructions are read to ensure the subject does not attempt to determine the token order before the timing of the task begins.*

6 **Dual Transfer task:** The subject is asked to transfer the tokens in order of value from highest to lowest whilst continuously reciting the alphabet as quickly as possible. *Again, the tokens must remain covered whilst the test instructions are read to ensure the subject does not start working out the token order before the test begins.*

VERBAL INSTRUCTIONS FOR EACH TASK

The subject is asked to sit facing the table, with the C3t set up in front of them. They should start with their hands placed on their legs.
The following instructions are given for each task:

1 **Baseline Transfer task:**
When I say "Go," using your non-dominant hand I want you to pick up each token individually, pass it to your dominant hand and put it in the container. I want you to start with the largest token, (the one furthest from you and work your way down to the smallest token which is closest to you). I want you to do this as quickly as possible and I will stop timing you after you have placed the last token into the container. If you drop a token and it falls or rolls outside of this area **(Indicate the outer edge of the C3t case to the subject)**, *please leave it and move onto your next token. If you drop the token and it falls on the surface in front of you* **(Indicate that this is within the foam of the C3t case)**, *you can pick it up and continue. I would like you to start with your hands on your legs. Do you have any questions? Ready? Go"*

2 **Baseline Value task**
"Using the values printed on this card, I want you to say aloud the highest value and work your way in decreasing order of value to the lowest value. I want you to do this as quickly as you can. Do you have any questions? Ready? Go"

3 **Complex Value task (optional)**
"Using the values printed on this card, I want you to say aloud the highest value and work your way in order to the lowest value. I want you to do this as quickly as you can. Do you have any questions? Ready? Go."

4 **Baseline Alphabet task (optional)**
"I would like you to recite the alphabet, pronouncing each letter, as quickly as possible. Do you have any questions? Ready? Go"

5 **Complex Transfer task**
(Keep the tokens covered using the Complex value card whilst reading the instructions) *"When I say "Go," using your non-dominant hand I want you to pick up each token individually, pass it to your dominant hand and put it in the container. I want you to transfer the tokens in order of value, starting with the highest value and ending with the lowest. I want you to do this as quickly as possible and I will stop timing you after you have released the last token into the container. If you drop a token and it falls or rolls outside of this area* **(Indicate the outer edge of the C3t case to the subject)**, *please leave it and move onto your next token. If you drop the token and it falls on the surface in front of you* **(Indicate that this is within the foam of the C3t case)**, *you can pick it up and continue. I would like you to start with your hands on your legs. Do you have any questions? Ready? Go"* **(Remove the Complex value card to reveal the tokens)**.

6 **Dual Transfer task (optional)**
(Keep the tokens covered using the empty Complex transfer task token tray whilst reading the instructions) "When I say "Go," using your non-dominant hand I want you to pick up each token individually, pass it to your dominant hand and put it in the container. I want you to transfer the tokens as quickly as possible in order of value, starting with the highest value and ending with the lowest value. Whilst doing this I want you to recite the alphabet as quickly as you can. If you finish the alphabet before you finish this transfer task, start reciting the alphabet again and keep doing this until you have placed the last token into the container. If you drop a token and it falls or rolls outside of this area **(Indicate the outer edge of the C3t case to the subject)**, please leave it and move onto your next token. If you drop the token and it falls on the surface in front of you **(Indicate that this is within the foam of the C3t case)**, you can pick it up and continue. I would like you to start with your hands on your legs. Do you have any questions? Ready? Go" **(Remove the complex transfer task token tray to reveal the tokens)**.

SCORING THE C3t

The time to complete the Baseline Transfer task, Complex Transfer task and dual transfer task is recorded in seconds. The researcher should start timing as soon as the subject is instructed to "Go" and stop as soon as the last token is released from the subject's fingers into the container.

The researcher should observe the subject whilst performing the C3t and record any of the following errors:

- o If the subject does not transfer the tokens correctly (from non-dominant to dominant) between their hands this is recorded as a **transfer error**. The assessor can remind the subject of the rules **once** by saying for example, 'remember to transfer the token between your hands' or 'remember to transfer the token from the highest value to the lowest.'
- o If the subject transfers the tokens in the wrong order this is recorded as a **rule error**.

If the subject drops a token and this falls outside of the C3t case, remind the subject to leave it and to continue with the next token. Record this as a token dropped out of reach (dropped tokens). If the token is dropped but the subject quickly retrieves it with little test disturbance, it should still be recorded as a dropped token error.

For each task, the time taken and any errors committed are recorded. Total task time is the primary meaningful scoring metric for the C3t. It is however also possible to calculate accuracy or produce performance cost metrics which can provide a more detailed indication of dual task performance in different populations.

Table 1 provides a summary of the variables that are generated during the performance of the C3t. It is possible to further derive composite measures that take into account time and error or to produce time percentage cost variables between the 3 levels of transfer tasks.

Table 1: C3t variables and scoring methods

Variable Name	Variable Description
Baseline Transfer dropped tokens	Number of tokens dropped or rolled out of reach in Baseline transfer task
Baseline Transfer rule errors	Number of rule errors committed in the Baseline transfer task
Baseline Transfer, transfer errors	Number of transfer errors committed in the Baseline transfer
Baseline Transfer time	Time taken (s) to complete the Baseline transfer task

Baseline Value correct values	Number of correct values (out of 8) in the Baseline Value task
Complex Value correct values (optional)	Number of correct values (out of 8) in the Complex Value task
Baseline Alphabet correct letters (optional)	Number of correct letters of the alphabet recited in the Baseline Alphabet task (only 1 recital of the full alphabet)
Complex Transfer dropped tokens	Number of tokens dropped or rolled out of reach in the complex transfer task
Complex Transfer rule errors	Number of rule errors committed in the complex transfer task
Complex Transfer, transfer errors	Number of transfer errors committed in the complex transfer task
Complex Transfer time	Time taken (s) to complete the complex transfer task
Dual Transfer dropped tokens	Number of tokens dropped or rolled out of reach in the dual transfer task
Dual Transfer rule errors	Number of rule errors committed in the dual transfer task
Dual Transfer, transfer errors	Number of transfer errors committed in the dual transfer task
Dual Transfer time	Time taken (in seconds) to complete the dual transfer task

FREQUENTLY ASKED QUESTIONS (FAQS)

How do I fold away the C3t?

Remove the container and place it into the holder located on the side crease of the C3t case. Next, place the tokens into the correct position on the correct tray. Stack the token trays and value cards in the same order they were setup and stretch the elastic band length way around the token trays to secure them in place. Fold end of the assessment case that holds the token trays to the centre, fold the final side of the case and push the poppers to secure it.

What if the subject drops a token on the floor?

If a token is dropped outside the C3t case or on the floor, remind the subject to leave it and continue with the next token. Record the number of tokens dropped out of reach.

What if the subject drops a token outside the C3t case but quickly retrieves it?

If the token is dropped just outside the container or on the subject's lap and the subject retrieves the token immediately this is still counted as as a 'dropped token'.

What if the subject starts transferring the tokens in the wrong order or forgets to transfer the tokens between their hands?

After the first error has been made, the assessor can remind the subject of the rules once by saying for example, 'remember to transfer the token between your hands' or 'remember to transfer the token from the highest value to the lowest.' Only one prompt is allowed. Any further errors should be recorded (along with the first error) as transfer error.

What if the subject forgets the alphabet or stops reciting the alphabet mid-way through the C3t dual task?

If the subject forgets the alphabet (after 5 seconds of silence), the assessor can repeat the last letter the subject recited. If this does not trigger a response then the researcher can recite the beginning letter of the alphabet, hinting for the subject to start again. Although the main aim of the C3t dual task is that the subject recites the alphabet whilst transferring the tokens, the assessor can prompt the subject with the alphabet as described above. If, after prompting as above, they fail to recite the alphabet (or start and then stop again, with 5 seconds of silence), the task should be stopped and it should be recorded that they are unable to complete the task.

Who can I contact if I have any questions?

You can contact the developers at the Centre for Trials Research, Cardiff University by email: ctr@cardiff.ac.uk

DATA COLLECTION SHEET

1 Baseline Transfer Task

Time taken (s)		Number of transfer errors	
Number of rule errors		Number of dropped tokens	

2 Baseline Value Task

Task attempted:		Yes		No
Task passed:		Yes		No

Tokens: Correct order
(mark each value recited correctly)

200 100 50 20 10 5 2 1

3 Complex Value Task

Task attempted:		Yes		No
Task passed:		Yes		No

Tokens: Correct order
(mark each value recited correctly)

90 82 71 49 35 17 6 3

4 Baseline Alphabet

Alphabet language:

Time taken (s) | Number of correct letters

Task attempted: ☐ Yes ☐ No

Task passed: ☐ Yes ☐ No

Alphabet: Correct order
(mark each letter recited correctly)

A	B	C	D	E	F	G	H	I	J
K	L	M	N	O	P	Q	R	S	T
U	V	W	X	Y	Z				

5 Complex Transfer Task

Time taken (s) | Number of transfer errors

Number of rule errors | Number of dropped tokens

Task attempted: ☐ Yes ☐ No

Task passed: ☐ Yes ☐ No

Tokens: Correct order
(mark each value transferred correctly)

200 100 50 20 10 5 2 1

6 Dual Transfer Task

Time taken (s)		Number of transfer errors	
Number of rule errors		Number of dropped tokens	
Number of correct letters			

Task attempted: ☐ Yes ☐ No

Task passed: ☐ Yes ☐ No

Tokens: Correct order
(mark each value transferred correctly)

90 82 71 49 35 17 6 3

Alphabet: Correct order
(mark each letter recited correctly during the task)

A ☐	B ☐	C ☐	D ☐	E ☐	F ☐	G ☐	H ☐	I ☐	J ☐
K ☐	L ☐	M ☐	N ☐	O ☐	P ☐	Q ☐	R ☐	S ☐	T ☐
U ☐	V ☐	W ☐	X ☐	Y ☐	Z ☐				

A ☐	B ☐	C ☐	D ☐	E ☐	F ☐	G ☐	H ☐	I ☐	J ☐
K ☐	L ☐	M ☐	N ☐	O ☐	P ☐	Q ☐	R ☐	S ☐	T ☐
U ☐	V ☐	W ☐	X ☐	Y ☐	Z ☐				

A ☐	B ☐	C ☐	D ☐	E ☐	F ☐	G ☐	H ☐	I ☐	J ☐
K ☐	L ☐	M ☐	N ☐	O ☐	P ☐	Q ☐	R ☐	S ☐	T ☐
U ☐	V ☐	W ☐	X ☐	Y ☐	Z ☐				

This work was supported in part by Medical Research Council Confidence in Concept Scheme, Health and Care Research Wales BRAIN unit and the Wellcome Trust ISSF funds. S Clinch was funded through REPAIR-HD which is funded from the European Union's Seventh Framework Programme under grant agreement n°602245 (http://www.repair-hd.eu/).
