# Supplementary material for: Objectively characterizing Huntington’s disease using a novel upper limb dexterity test
Source: J Neurol. 2021 Feb 8;268(7):2550–9. doi: 10.1007/s00415-020-10375-8 (PMC7868671; doi:10.1007/s00415-020-10375-8)
Supplement: Supplementary file 2 — Supplementary file2 Supplementary material 2: The coefficients reported from a LASSO regression when predicting clinical scores using either the BTT or CTT C3t scores, taking into consideration the site at which the data was collected at and the version of the C3t that was performed. The lower the magnitude of a coefficient the less impact the relevant variable has on the model. Coefficients are reported as mean and standard deviation (SD) to reflect the variation across the cross-validation folds within the regression analyses. Only the site and C3t version with the highest coefficient are reported to determine how much site and test version contributed to the regression model. Whilst some site and test version coefficients were non-zero, their magnitude was significantly smaller and more variable than the C3t time score coefficients, suggesting they had limited impact on the model relative to the C3t (DOCX 24 KB) [file 415_2020_10375_MOESM2_ESM.docx]

Supplementary material 2: The coefficients reported from a LASSO regression when predicting clinical scores using either the BTT or CTT C3t scores, taking into consideration the site at which the data was collected at and the version of the C3t that was performed. The lower the magnitude of a coefficient the less impact the relevant variable has on the model. Coefficients are reported as mean and standard deviation (SD) to reflect the variation across the cross-validation folds within the regression analyses. Only the site and C3t version with the highest coefficient are reported to determine how much site and test version contributed to the regression model. Whilst some site and test version coefficients were non-zero, their magnitude was significantly smaller and more variable than the C3t time score coefficients, suggesting they had limited impact on the model relative to the C3t.

|  | **LASSO Regression coefficients when predicting clinical scores using the BTT** | | | | | | **LASSO Regression coefficients when predicting clinical scores using the CTT** | | | | | |
| --- | --- | --- | --- | --- | --- | --- | --- | --- | --- | --- | --- | --- |
|  | **BTT coefficient** | | **Max coefficient across all sites** | | **Max coefficient across C3t test versions** | | **CTT coefficient** | | **Max coefficient across sites** | | **Max coefficient across C3t test versions** | |
|  | **Mean** | **SD** | **Mean** | **SD** | **Mean** | **SD** | **Mean** | **SD** | **Mean** | **SD** | **Mean** | **SD** |
| **Composite Unified Huntington’s Disease Rating Scale (cUHDRS)** | -1.53 | 0.91 | 0.00 | 0.00 | 0.00 | 0.00 | -1.61 | 0.84 | 0.00 | 0.00 | 0.00 | 0.00 |
| **Total Motor Score (TMS)** | 40.60 | 3.13 | 1.06 | 1.15 | -0.01 | 0.05 | 35.98 | 2.99 | -5.83 | 2.10 | -0.01 | 0.03 |
| **Symbol Digit Modalities Test (SDMT)** | -24.70 | 3.31 | 3.51 | 2.16 | -0.13 | 0.49 | -21.28 | 2.26 | 3.87 | 2.10 | -0.13 | 0.52 |
| **Stroop Word Reading Test (SWRT)** | -40.32 | 4.79 | 9.35 | 4.88 | -0.13 | 0.28 | -39.46 | 3.19 | 9.00 | 4.49 | 0.31 | 0.63 |
| **Total Functional Capacity (TFC)** | 0.00 | 0.00 | 0.00 | 0.00 | 0.00 | 0.00 | 0.00 | 0.00 | 0.00 | 0.00 | 0.00 | 0.00 |
