# Supplementary material for: Objectively characterizing Huntington’s disease using a novel upper limb dexterity test
Source: J Neurol. 2021 Feb 8;268(7):2550–9. doi: 10.1007/s00415-020-10375-8 (PMC7868671; doi:10.1007/s00415-020-10375-8)
Supplement: Supplementary file 3 — Supplementary file3 Supplementary material 3: The number of participants recruited across each sub-study and disease stage (DOCX 21 KB) [file 415_2020_10375_MOESM3_ESM.docx]

Supplementary material 3: The number of participants recruited across each sub-study and disease stage.

|  | **Number of participants recruited across each sub-study (n)** | | | |  |
| --- | --- | --- | --- | --- | --- |
| **Disease Stage** | **PACE-HD** | **TRIDENT** | **CAPIT-HD2** | **NOVELFA-C3T** | **TOTAL** |
| **Pre-manifest** | 0 | 3 | 3 | 10 | 16 |
| **TFC Stage 1** | 9 | 5 | 22 | 3 | 39 |
| **TFC Stage 2** | 11 | 5 | 21 | 6 | 43 |
| **TFC Stage 3** | 0 | 2 | 3 | 2 | 7 |
| **TOTAL** | 20 | 15 | 49 | 21 | N/A |
